# Atezolizumab plus bevacizumab combination enables an unresectable hepatocellular carcinoma resectable and links immune exclusion and tumor dedifferentiation to acquired resistance

**DOI:** 10.1186/s40164-021-00237-y

**Published:** 2021-08-16

**Authors:** Yulei Wang, Li-Chun Lu, Yinghui Guan, Ming-Chih Ho, Shan Lu, Jessica Spahn, Chih-Hung Hsu

**Affiliations:** 1 Oncology Biomarker Development, Genentech, South San Francisco, USA; 2 Department of Oncology, National Taiwan University Hospital, Taipei, Taiwan; 3 Department of Surgery, National Taiwan University Hospital, Taipei, Taiwan; 4 Graduate Institute of Oncology, National Taiwan University College of Medicine, Taipei, Taiwan; 5 Department of Medical Oncology, National Taiwan University Cancer Center, Taipei, Taiwan; 6 Product Development Oncology, Genentech, South San Francisco, USA

**Keywords:** Atezolizumab, Bevacizumab, Hepatocellular carcinoma, Immune exclusion, PD-L1

## Abstract

We reported a patient with unresectable hepatocellular carcinoma (HCC) who initially received 15 cycles of atezolizumab plus bevacizumab combination and had best tumor response of partial response, but later experienced disease progression. After subsequent surgical resection, the patient enjoyed long-term disease-free status at the last follow-up 19 months after surgery. By investigating paired tumor tissues (pretreatment and post-progression samples) with immunohistochemistry, multiplex immunofluorescence, RNA sequencing, and DNA sequencing, we explored the dynamic changes in the tumor microenvironment (TME) and potential mechanisms underlying acquired resistance to the combination. In the post-progression HCC tissue compared with the baseline tissue, the expression of PD-L1 in tumor-infiltrating immune cells and the abundance of CD8^+^ T cells in the tumor area had decreased, and an immune-excluded TME had emerged. Transcriptomic analysis revealed a gene expression signature representing progenitor/hepatoblast features in the post-progression tumor tissue, with an increased expression of imprinted genes and decreased expression of cytochrome P450 family genes. Finally, tumor mutational burden and MHC class I expression in tumor cells were both increased in the post-progression tissue, suggesting that neoantigen depletion or loss-of-antigen presentation were unlikely causes of acquired resistance in this patient. Atezolizumab plus bevacizumab combination therapy enabled our patient to receive hepatectomy and achieve long-term remission. A comparison of paired tumor tissues suggested that immune-excluded TME and tumor dedifferentiation may have contributed to acquired resistance to the combination.

**Supplementary Information:**

The online version contains supplementary material available at 10.1186/s40164-021-00237-y.

Atezolizumab, an PD-L1 monoclonal antibody, plus bevacizumab, an anti-VEGF monoclonal antibody, (atezo/bev) has become a new standard-of-care as the first-line therapy for patients with advanced hepatocellular carcinoma (HCC) [1]. Here, we describe a patient with unresectable HCC who was able to receive tumor resection after an initial partial response to atezo/bev and progressed after 40 weeks of treatment.

A 63-year-old man was diagnosed with intermediate-stage HCC and initially received transarterial chemoembolization. His disease progressed with an enlarged right hepatic tumor and the occurrence of multiple new hepatic tumors. He was enrolled in a clinical trial and received atezo/bev [2]. Small hepatic tumors responded significantly and the huge right hepatic tumor also became smaller. However, after 40 weeks of atezo/bev, follow-up computed tomography (CT) scans revealed progression of the huge tumor. Surgery with right hepatectomy was performed 1 month after the 15th cycle of the atezo/bev. The patient received active surveillance only after the surgery. At the last follow-up, at 19 months post-operation, CT scans indicated complete remission (Fig. 1).

By investigating the pretreatment and post-progression tumor tissues, we explored the dynamic changes in the tumor microenvironment (TME) and potential mechanisms underlying acquired resistance to atezo/bev. Immunohistochemistry (IHC) analysis revealed that the expression of PD-L1 in tumor-infiltrating immune cells and the abundance of CD8^+^ T cells in the tumor area had decreased. The multiplex immunofluorescence assay also revealed reduced density of CD8^+^ T cells in the tumor center but increased density in the surrounding stroma in the tissue obtained at the time of disease progression, indicating an immune-excluded phenotype. In addition, quantification based on a digital pathology algorithm indicated that expression of the activated fibroblast marker, FAP, was significantly increased in the disease progression sample, whereas the blood vasculature marker, CD31, was decreased. MHC class I expression on tumor cells was increased in the post-progression tissue (Fig. 2A).

A gene expression signature representing progenitor/hepatoblast features was found to be increased at the time of disease progression, indicating that tumor cells might have undergone dedifferentiation process to acquire resistance to atezo/bev. Supporting this hypothesis, a significant re-expression of several imprinted genes was also found in tissue collected upon disease progression, and genes of the cytochrome P450 family, which are typically highly expressed in well-differentiated hepatocytes, were found to be downregulated in the post-progression tumor tissue (Fig. 2B). Consistent with the IHC findings, gene expression analysis confirmed the high expression of PD-L1 (CD274) and T effector signature in the pretreatment sample and decreased expression of them in the disease progression sample (Fig. 2B). Whole-exome sequencing showed the tumor mutational burden (TMB) increased from 5.7 mutations/Mb at baseline to 9.3 mutations/Mb at the time of disease progression. Shared somatic mutations in both pre- and post-treatment tissues or new somatic mutations detected only in the post-treatment tissues were identified (Additional file 1).

## Discussion

Effective systemic therapy may serve as a “conversion” therapy and enable patients with unresectable HCC to become amenable to curative locoregional therapies [3, 4]. Anti-angiogenesis plus immune-checkpoint blockade substantially improved therapeutic efficacy in many solid tumors, including advanced HCC [5–7]. Atezo/bev might also have a strong impact on the treatment of localized HCC.

We analyzed HCC tissues before and after atezo/bev treatment. The expression of PD-L1 in tumor-infiltrating immune cells and the abundance of CD8^+^ T cells in the tumor area had decreased, and an immune-excluded TME had emerged in the post-progression HCC tissue [8]. These findings agree with a study reporting that high expression of PD-L1 and T effector signature may predict better outcome in patients with advanced HCC treated with atezo/bev [9]. The observations of increased activated stroma (FAP) coincides with our previous findings in ovarian cancer showing that TGFβ signaling as an important mediator of T cell exclusion by activating fibroblasts and inducing extracellular matrix production [10]. Our transcriptomic analysis also revealed several tumor-intrinsic features, related to tumor dedifferentiation, may be associated with acquired resistance to atezo/bev therapy. In addition, TMB and MHC class I expression on tumor cells were both increased at the time of disease progression, suggesting that loss-of-antigen presentation and neoantigen depletion were unlikely mechanisms driving acquired resistance in this patient.

Although PD-L1 expression and TMB were reported to be associated with responses to immune checkpoint blockade in multiple cancer types, their roles as predictive biomarkers of immune checkpoint blockade have not been defined in many other cancer types. A study showed CD8^+^ T cells infiltration was correlated with PD-L1 expression, but not with TMB, further echoing our findings in this case [11]. Future biomarker studies, employing multi‑omics signatures to characterize TME, are warranted to identify predictors for immune checkpoint treatment response [12].

In summary, atezo/bev therapy enabled our patient to receive hepatectomy and achieve long-term remission. The potential resistance mechanisms of the combination therapy identified in our study warrant further investigations.

**Fig. 1:**
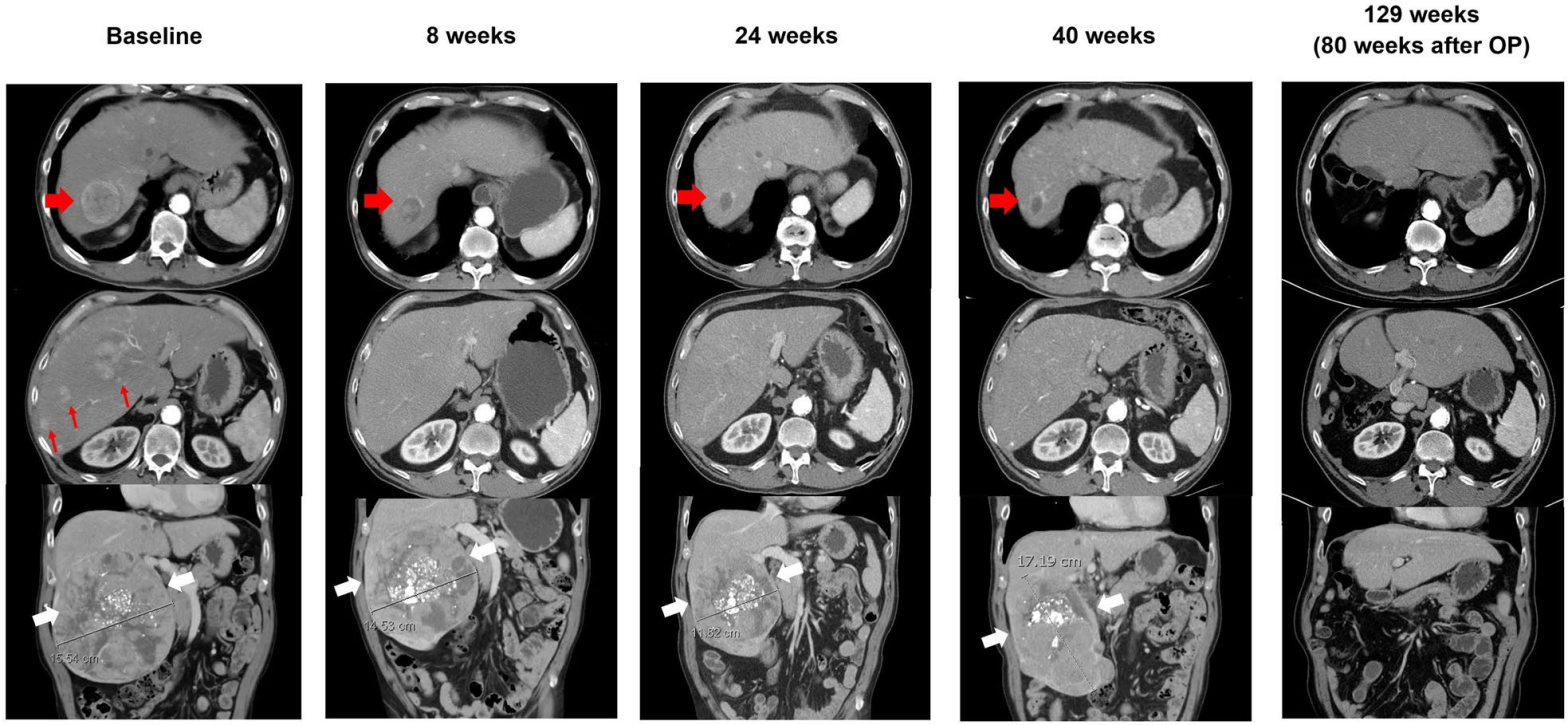
Changes in tumor burden after atezolizumab plus bevacizumab combination therapy. Representative images at baseline (before atezolizumab plus bevacizumab treatment), at different times after systemic therapy initiation and after right hepatectomy operation (OP). The red arrowhead indicates the target lesion, which shrank significantly after treatment; red arrows indicate several nontarget lesions, which resolved completely thereafter; and white arrowheads indicate the huge tumor, which was treated with transarterial chemoembolization before systemic therapy and was resected upon progression

**Fig. 2:**
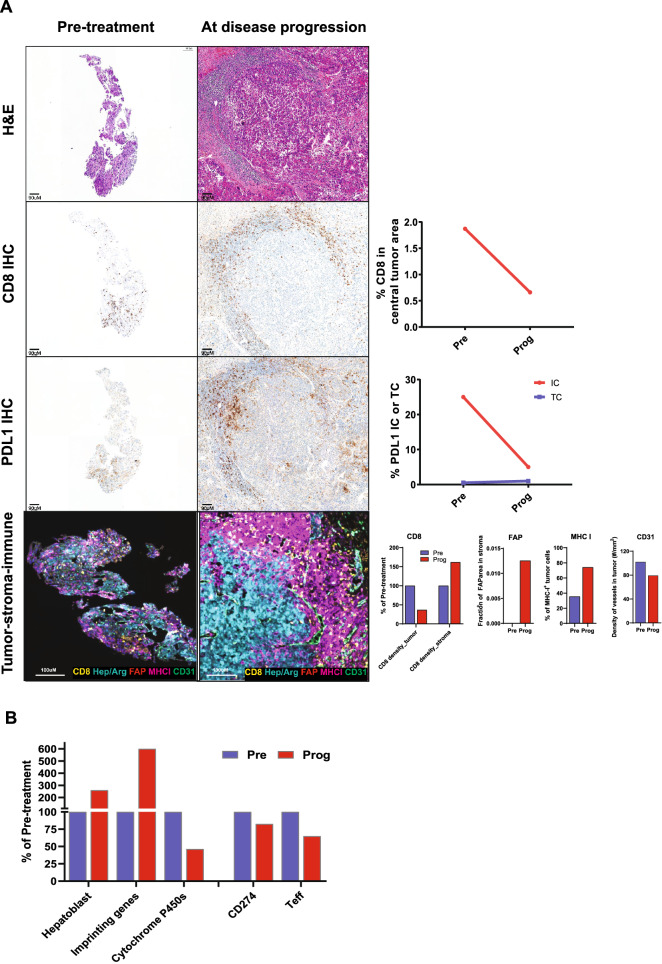
Dynamic changes of the tumor microenvironment and tumor-intrinsic features in tumor tissues obtained pre-treatment of atezolizumab plus bevacizumab combination therapy and post-progression. **A** Top row: Hematoxylin and eosin staining; Second row: CD8 immunohistochemistry (IHC) revealed that the percentage of CD8^+^ T cells in the central tumor area was also decreased in the post-progression tumor tissue. Third row: PD-L1 IHC showing decreased PD-L1 expression in tumor-infiltrating immune cells (ICs) after disease progression but no change in expression in tumor cells (TCs). Bottom row: Multiplex immunofluorescence assay and the digital pathology algorithm showed an immune-excluded pattern in the post-progression tumor tissue. CD8^+^ T cells were surrounded outside the tumor area, which was defined by HepPar-1 and arginase-1 (Hep/Arg) positivity. A digital pathology algorithm showed the expression of CD8 decreased in tumor area but increased in peritumor stroma. The activated fibroblast marker (FAP) was increased while the blood vasculature marker, CD31, was decreased in the disease progression sample. MHC class I expression in TCs was increased in the tissue collected at the time of disease progression compared with that collected at baseline. **B** RNA sequencing revealed increased expression of hepatoblast/progenitor signature (i.e., BUB1, DLGAP5, DUSP9, E2F5, IGSF1, NLE1, and RPL10A) and imprinted genes (i.e., DLK1, PEG3, and ZIM2), decreased expression of cytochrome p450 genes (i.e., ADH1C, CYP1A2, CYP2A6, CYP2B6, CYP2C8, CYP2C9, and CYP3A4), and decreased expression of PD-L1 mRNA (CD274) and an T effector signature (GZMB, CXCL9, and PRF1) in the post-progression tissue

## Supplementary Information

**Additional file 1: Materials and Methods; Table S1.** Summary of mutations identified in tumor tissues collected at baseline and at disease progression.

## Data Availability

Not applicable.

## References

[1] Finn RS, Qin S, Ikeda M, Galle PR, Ducreux M, Kim TY (2020). Atezolizumab plus bevacizumab in unresectable hepatocellular carcinoma. N Engl J Med.

[2] Lee MS, Ryoo BY, Hsu CH, Numata K, Stein S, Verret W (2020). Atezolizumab with or without bevacizumab in unresectable hepatocellular carcinoma (GO30140): an open-label, multicentre, phase 1b study. Lancet Oncol.

[3] Shao YY, Ho MC, Cheng AL, Hsu CH (2014). Long-term disease-free survival achieved by anti-angiogenic therapy plus surgery in a hepatocellular carcinoma patient with extensive liver involvement and lung metastases. J Formos Med Assoc.

[4] Lu LC, Shao YY, Chan SY, Hsu CH, Cheng AL (2014). Clinical characteristics of advanced hepatocellular carcinoma patients with prolonged survival in the era of anti-angiogenic targeted-therapy. Anticancer Res.

[5] Yi M, Jiao D, Qin S, Chu Q, Wu K, Li A (2019). Synergistic effect of immune checkpoint blockade and anti-angiogenesis in cancer treatment. Mol Cancer.

[6] Liu TH, Shao YY, Hsu CH (2021). It takes two to tango: breakthrough advanced hepatocellular carcinoma treatment that combines anti-angiogenesis and immune checkpoint blockade. J Formos Med Assoc.

[7] Hack SP, Zhu AX, Wang Y (2020). Augmenting anticancer immunity through combined targeting of angiogenic and PD-1/PD-L1 pathways: challenges and opportunities. Front Immunol.

[8] Chen DS, Mellman I (2017). Elements of cancer immunity and the cancer-immune set point. Nature.

[9] Zhu AX, Guan Y, Abbas AR, Koeppen H, Lu S, Hsu CH (2020). Abstract CT044: Genomic correlates of clinical benefits from atezolizumab combined with bevacizumab vs. atezolizumab alone in patients with advanced hepatocellular carcinoma (HCC). Can Res.

[10] Desbois M, Udyavar AR, Ryner L, Kozlowski C, Guan Y, Dürrbaum M (2020). Integrated digital pathology and transcriptome analysis identifies molecular mediators of T-cell exclusion in ovarian cancer. Nat Commun.

[11] Chen Y, Wang Y, Luo H, Meng X, Zhu W, Wang D (2020). The frequency and inter-relationship of PD-L1 expression and tumour mutational burden across multiple types of advanced solid tumours in China. Exp Hematol Oncol.

[12] Liu F, Qin L, Liao Z, Song J, Yuan C, Liu Y (2020). Microenvironment characterization and multi–omics signatures related to prognosis and immunotherapy response of hepatocellular carcinoma. Exp Hematol Oncol.

