# Supplementary material for: Atezolizumab plus bevacizumab combination enables an unresectable hepatocellular carcinoma resectable and links immune exclusion and tumor dedifferentiation to acquired resistance
Source: Exp Hematol Oncol. 2021 Aug 16;10:45. doi: 10.1186/s40164-021-00237-y (PMC8365942; doi:10.1186/s40164-021-00237-y)
Supplement: Supplementary file 1 — Additional file 1: Materials and Methods; Table S1. Summary of mutations identified in tumor tissues collected at baseline and at disease progression. [file 40164_2021_237_MOESM1_ESM.pdf]

Supplementary data

Materials and Methods

Atezolizumab plus bevacizumab combination therapy

GO30140 is an open-label, multicenter, multiarm, phase 1b study evaluating atezolizumab-based combination therapies in multiple malignant diseases. Group A was a single arm cohort enrolling patients with unresectable HCC who had received no previous systemic therapy and were not amenable to curative treatment. Other key eligibility criteria included aged 18 years and older, diagnosis of HCC by histology, cytology, or clinical criteria per American Association for the Study of Liver Disease, measurable disease according to Response Evaluation Criteria in Solid Tumors version 1.1 (RECIST 1.1), Eastern Cooperative Oncology Group performance score of 0 or 1, adequate hematological and organ functions, and informed consent. Enrolled patients received atezolizumab (1200 mg intravenously every 3 weeks) plus bevacizumab (15 mg/kg intravenously every 3 weeks) continuously until loss of clinical benefit or unacceptable toxicity. Tumor assessments were done every 8 weeks for the first year and every 12 weeks thereafter until patient death, disease progression, or initiation of further systemic cancer therapy. The results of GO30140 have been previously reported.[1]

Collection of tumor samples

The tumor tissue at baseline was collected through computed tomography-guided percutaneous biopsy of the huge hepatic tumor (18 cm in diameter) on the right lobe 3 weeks prior to the start of atezolizumab plus bevacizumab combination therapy. The tumor tissue at disease progression was collected from the huge hepatic tumor during the salvage surgery with right hepatectomy 1 month after the final treatment of atezolizumab plus bevacizumab combination therapy. All clinical samples had relevant institutional review board approval, and written informed consent was obtained from the patient.

Immunohistochemical assessments

PD-L1 expression was assessed using immunohistochemistry (IHC) with a PD-L1 IHC SP263 assay (Ventana Medical Systems; Tucson, AZ, USA). PD-L1 expression in tumor cells (TCs) and tumor-infiltrating immune cells (ICs) were reported as the TC and IC score, respectively. TC score: percentage of total tumor cells expression PD-L1; IC: tumor-infiltrating immune cells expressing PD-L1 as a percentage of tumor area. IHC of CD8 was performed using anti-CD8 [144B] (Abcam, Cambridge, MA, USA).

Multiplex immunofluorescence

A 5-plex immunofluorescence (IF) assay was performed on a Ventana BenchMark Ultra automated staining instrument (Ventana Medical Systems).[2] This tumor–stroma–immune context panel contained five biomarkers: HepPar1 (OCH1E5, Cell Marque)/Arginase (CL0186, NovasBio), CD8 (SP239, Abcam), fibroblast activation protein (FAP) (SP325, Abcam), CD31 (EPR3095, Abcam), and MHC class I (EP1395Y, Abcam). Image analysis, by quantifying cells with either uniquely stained or concurrently stained markers within the regions of interest (ROIs), was performed on scanned images as previously described.[3] The DP algorithm was used to report statistical metrics regarding the density of objects and their spatial interrelationships in automatically computed ROIs.

RNA and DNA sequencing assessments

Macrodissection was performed on formalin-fixed, paraffin-embedded tumor tissues to enrich the tumor percentage to >50%. RNA sequencing was performed using TruSeq RNA Access technology (Illumina®). Whole-exome sequencing was performed using SureSelectXT Exome Target Enrichment System (Agilent) for Illumina Sequencing, V5 DNA baits. Exome-enriched libraries were PCR amplified to

complete the Illumina adaptor and then sequenced to 100X coverage (2x100 PE) using Illumina HiSeq 2500, Rapid Run system, with on-board cluster generation, V1 chemistry.

References

1. Lee MS, Ryoo BY, Hsu CH, Numata K, Stein S, Verret W, et al. Atezolizumab with or without bevacizumab in unresectable hepatocellular carcinoma (GO30140): an open-label, multicentre, phase 1b study. *Lancet Oncol* 2020;21:808-20.
2. Zhang W, Hubbard A, Jones T, Racolta A, Bhaumik S, Cummins N, et al. Fully automated 5-plex fluorescent immunohistochemistry with tyramide signal amplification and same species antibodies. *Lab Invest* 2017;97:873-85.
3. Racolta A KM, Giltnane J, Hubbard A, Zhang H, Matei M, Baumann J, et al. Quantification of tumor-stroma-immune contexture by multiplex fluorescent immunohistochemistry and whole-slide digital image analysis. Paper presented at: 34th Annual Meeting & Pre-Conference Programs of the Society for Immunotherapy of Cancer (SITC 2019): part 12019; National Harbor, MD, USA. 6-10 November 2019.

Table S1. Summary of mutations identified in tumor tissues collected at baseline and at disease progression

Symbol	Mutation	Allelic frequency Pre-treatment	Allelic frequency At disease progression
Mutations detected in both pre-treatment and at disease progression tumor tissues			
TP53	251 I/F	0.1	0.68
CTNNB1	34 G/V	0.15	0.25
PCLO	1038 A/S	0.12	0.22
FAT4	3425 F/I	0.17	0.43
NCOR2	2234 S/F	0.1	0.37
MECOM	596 T/N	0.13	0.24
GRIN2A	680 F/L	0.23	0.33
STAT5B	555 W/C	0.15	0.26
Mutations detected from tumor tissue at disease progression only			
KMT2C	1227 H/R	NA	0.3
CARD11	141 Q/H	NA	0.32
HGF	380 I/F	NA	0.27
SOS1	170 K/E	NA	0.42
XPO1	662 N/T	NA	0.39
PAX3	289 L/I	NA	0.38
FGF23	182 E/V	NA	0.18
